# Combining Hyperspectral Reflectance Indices and Multivariate Analysis to Estimate Different Units of Chlorophyll Content of Spring Wheat under Salinity Conditions

**DOI:** 10.3390/plants11030456

**Published:** 2022-02-07

**Authors:** Salah El-Hendawy, Yaser Hassan Dewir, Salah Elsayed, Urs Schmidhalter, Khalid Al-Gaadi, ElKamil Tola, Yahya Refay, Muhammad Usman Tahir, Wael M. Hassan

**Affiliations:** 1 Department of Plant Production, College of Food and Agriculture Sciences, King Saud University, KSA, P.O. Box 2460, Riyadh 11451, Saudi Arabia; ydewir@ksu.edu.sa (Y.H.D.); refay@ksu.edu.sa (Y.R.); mtahir@ksu.edu.sa (M.U.T.); 2 Agricultural Engineering, Evaluation of Natural Resources Department, Environmental Studies and Research Institute, University of Sadat City, Sadat City 32897, Egypt; salah.emam@esri.usc.edu.eg; 3 Chair of Plant Nutrition, Department of Plant Sciences, Technical University of Munich, Emil-Ramann-Str. 2, D-85350 Munich, Germany; schmidhalter@wzw.tum.de; 4 Department of Agricultural Engineering, Precision Agriculture Research Chair (PARC), College of Food and Agriculture Sciences, King Saud University, KSA, P.O. Box 2460, Riyadh 11451, Saudi Arabia; kgaadi@ksu.edu.sa (K.A.-G.); etola@ksu.edu.sa (E.T.); 5 Department of Agricultural Botany, Faculty of Agriculture, Suez Canal University, Ismailia 41522, Egypt; wmohamed@su.edu.sa

**Keywords:** content, concentration, different algorithm forms, non-destructive assessment, PLSR, phenotyping, remote sensing, SMLR

## Abstract

Although plant chlorophyll (Chl) is one of the important elements in monitoring plant stress and reflects the photosynthetic capacity of plants, their measurement in the lab is generally time- and cost-inefficient and based on a small part of the leaf. This study examines the ability of canopy spectral reflectance data for the accurate estimation of the Chl content of two wheat genotypes grown under three salinity levels. The Chl content was quantified as content per area (Chl _area_, μg cm^−2^), concentration per plant (Chl _plant_, mg plant^−1^), and SPAD value (Chl _SPAD_). The performance of spectral reflectance indices (SRIs) with different algorithm forms, partial least square regression (PLSR), and stepwise multiple linear regression (SMLR) in estimating the three units of Chl content was compared. Results show that most indices within each SRI form performed better with Chl _area_ and Chl _plant_ and performed poorly with Chl _SPAD_. The PLSR models, based on the four forms of SRIs individually or combined, still performed poorly in estimating Chl _SPAD_, while they exhibited a strong relationship with Chl _plant_ followed by Chl _area_ in both the calibration (Cal.) and validation (Val.) datasets. The SMLR models extracted three to four indices from each SRI form as the most effective indices and explained 73–79%, 80–84%, and 39–43% of the total variability in Chl _area_, Chl _plant_, and Chl _SPAD_, respectively. The performance of the various predictive models of SMLR for predicting Chl content depended on salinity level, genotype, season, and the units of Chl content. In summary, this study indicates that the Chl content measured in the lab and expressed on content (μg cm^−2^) or concentration (mg plant^−1^) can be accurately estimated at canopy level using spectral reflectance data.

## 1. Introduction

The greatest challenge for the agriculture sector in arid and semiarid regions is the scarcity of freshwater, which is increasing year after year due to population rise and the negative impacts of global warming. The use of non-conventional water resources such as drainage water, recycled water, and saline groundwater has been suggested as an alternative source of extraordinary interest to ensure the sustainable production of food crops in these regions [1,2,3]. However, continuous use of these water resources for irrigation, with a significant reduction in the annual rainfall, generates salinity stress problems. Exposure of plants to salinity stress leads to a significant reduction in their growth and potential yield due to the negative impacts of the different sub-components of salinity stress (osmotic stress, ionic imbalance, ion toxicity, and oxidative stress) on several plants’ physiological and biochemical processes [4,5,6,7]. Therefore, various agronomic approaches were applied to minimize these negative impacts of salinity stress on crop growth and productivity [6,8,9]. However, the availability of salt-tolerant genotypes for farmers still remains the more realistic and economical approach for sustainable crop production under salinity conditions; they reduce dependence on costlier agronomic approaches and are feasible to apply on a large scale. To develop the salt tolerance of genotypes, understanding the salt tolerance mechanisms that depend on physiological and photosynthetic indicators is a very important step in achieving this objective. This is because most of these indicators reflect the response of plants to salt stress at the levels of organ, tissue, and cellular, and therefore they make the evaluation of salt tolerance between genotypes more effective [5,6,10,11,12].

The sub-components of salinity stress interact together and lead to an increase in the level of reactive oxygen species (ROS) in plant cells, which in turn leads to accelerating chlorophyll (Chl) degradation or reduces Chl synthesis [13,14]. Previous studies reported that the reduction in leaf Chl content was greater in salt-sensitive genotypes than in salt-tolerant ones [6,7,11]. Thus, accurate estimation of leaf Chl content is quite useful in improving the salt tolerance of genotypes in breeding programs because it can be used as an effective screening criterion for discriminating the salt tolerance among genotypes.

Although the assessment of leaf Chl content through the traditional laboratory methods is very accurate, on the other side it is destructive, expensive, time-consuming, laborious, and often inappropriate for tracking the dynamic changes in Chl content when the measurements are made for a large number of genotypes at a large scale. The Chl meter devices such as SPAD-502 can be used to estimate the leaf Chl content for a large number of genotypes in an expeditious and non-destructive manner. Several previous studies have reported that the SPAD numerical values showed strong relationships with the leaf Chl content under both normal and stress conditions [15,16,17]. However, the main drawback of the traditional and SPAD methods is that they provide information on the state of the Chl content based on a single leaf or part of leaf measurements and disregard the vertical heterogeneity in the Chl content within a canopy. Therefore, they cannot reveal the actual status of Chl content of the whole plant canopy. Consequently, assessment of Chl content of a plant canopy using a hyperspectral remote sensing tool has recently received considerable attention as this tool has the ability to address the aforementioned drawbacks associated with SPAD and laboratory methods.

The ground-based hyperspectral reflectance tool has enabled us to capture the canopy spectral reflectance in the full range of the spectrum (400–2500 nm), and therefore it can provide important information on several canopy biophysical and biochemical characteristics. For example, the spectral reflectance from the canopy in the range of the visible spectrum (VIS, 400–700 nm), mainly in the blue (420–470 nm) and red (640–680 nm) regions, depends on the concentrations of leaf pigments, especially Chl content. The spectral reflectance in both regions is low with a peak at the green region (520–580 nm) when the leaf Chl concentration is high and vice versa [18,19,20,21]. Canopy reflectance in the red-edge region (RER, 680–750 nm) were also found to be closely related to the leaf Chl content in a wide range of its variation and therefore the information on this region of the spectrum provides a useful indicator of canopy Chl content [22,23,24,25,26]. Previous studies have also found that the spectral reflectance in the near-infrared (NIR) region, particularly the wavelengths around 750–900 nm, could provide an accurate estimation of Chl levels in plant [23,27,28]. Importantly, because the wavelengths in the NIR and shortwave-infrared (SWIR) regions of the spectrum ignore colour information in the background, spectral reflectance in both regions could also be used to provide useful information for canopy Chl estimation. Zou et al. [29,30] reported that the spectral reflectance at the wavelength region of 900–1700 nm can effectively reflect the Chl distribution map in cucumber plants. Therefore, this close relationship between canopy spectral reflectance and Chl content in plants can be exploited to develop an accurate, non-destructive, and rapid tool for Chl measurements.

To employ the spectral reflectance data collected from the canopy for estimating the leaf Chl content, different spectral reflectance-based indices (SRIs) have been developed by incorporating two or more wavelengths in a simple mathematical formula. Several SRIs have recently received considerable attention and have been found to be effective in assessing the status of Chl content in plants. The wavelengths in the formula of SRIs are usually built in different forms such as normalized difference (ND), modified normalized difference (MND), simple difference (SD), simple ratio (SR), modified simple ratio (MSR), or integration of more forms [28,31,32,33,34,35,36]. However, some forms of SRIs are effective for estimating plant traits than others because they are insensitive to a plants’ structural properties [18,28]. For example, the different normalized difference vegetation indices (NDVIs), which are in ND form and combine different wavelengths derived from the red region (670–680 nm) and the NIR region (750–900 nm), were not effective in assessing detailed canopy Chl due to their saturation at a high canopy Chl concentration (45 μg/cm^2^) and high leaf area index (LAI > 3) [21,23,26]. Wu et al. [19] found that the SRIs in MSR and MND-SR forms were more efficient than ND and SR in estimating the Chl content of maize plants because the former forms reduce the saturation effect. The capacity of SRIs for accurately estimating the Chl content was not only dependent on their form, but also on the number and type of wavelengths incorporated in their formula. For example, although the Modified Datt (MDATT) index (R_721_ − R_744_)/(R_721_ − R_714_) is in MND form, it was more effective than SD and SR in estimating Chl content over several plant species and at various growth stages. This is because this index incorporates three wavelengths from the RER, which are effective in eliminating the effects of leaf surface properties on the spectral reflectance [28,37]. Although the SRIs in either SR (R_750_/R_700_) or ND form (R_750_ − R_705_)/(R_750_ + R_705_) incorporated the most sensitive wavelength (700 nm) for Chl content, both indices showed a weak correlation with the Chl content when applied across a wide range of species. However, when this wavelength was combined with a third wavelength and incorporated in modified form (R_NIR_ − R_705_)/(R_NIR_ + R_705_ − 2R_445_), this new algorithm showed good performance in Chl estimation across a wide range of species [31,36]. Therefore, several studies have still worked to develop the algorithms of SRIs and define the sensitive wavelengths that would be efficient to eliminate the effects of leaf surface properties and saturation, if the estimation of absolute Chl content is desired. Consequently, to accurately estimate Chl content under different environmental conditions, including crop types, phenological stages, and levels of stress, using simple forms of SRIs, most published algorithms for SRIs need to be further validated, or new ones need to be developed. If the simple algorithms of SRI are accepted to be effective for the accurate estimation of leaf Chl content over a wide range of environmental conditions, developing a new lightweight device for remotely estimating total Chl content at the whole plant canopy level is possible and the limitations of the aforementioned SPAD meter could be overcome.

Commonly, Chl content is expressed either as a unit of mass (g plant^−1^) or a unit of area (μg cm^−2^). As both units are often used interchangeably in the relevant literature, the choice of the best algorithms of SRI for remotely estimating Chl content based on both units of Chl appears to be important. Grossman et al. [38] reported that the selection of the best wavelengths incorporated in the algorithms of SRI using stepwise multiple linear regression (SMLR) depends on the unit expression of the chemical data. Datt [39] found that the expression of Chl in plants using the unit of area is more suitable than using the unit of mass for remotely estimating the total Chl in plants. Consequently, comparing the performance of different forms or algorithms of SRIs for the accurate estimation of Chl in plants, based on different units of expression, is an important task that allows compatible lab measurements of Chl, with remotely sensed canopy reflectance at the canopy level scale.

In general, the performance of SRIs in the estimation of various plant traits is often inconsistent when measured across genotypes, years, sites, and phenological stages, as almost all of them include only two to three wavelengths [40,41,42,43]. Additionally, because the Chl content and LAI have similar influences on the spectral reflectance of the canopy, particularly at the wavelengths of 550 and 750 nm, these limited wavelengths influence the performance of SRIs when they are used individually to uncouple the combined effect of both traits [33]. Therefore, previous studies have reported that the performance of SRIs in the estimation of plant traits could be improved by coupling several of them with a proper multivariate regression method. Partial least squares regression (PLSR) and SMLR are the most multivariate methods in spectral studies and have become a popular approach for various plant traits estimation. Several studies have demonstrated that the two methods coupled with SRIs can achieve accurate estimation of different plant traits [37,41,42,44,45,46,47,48,49]. For example, Atzberger et al. [44] reported that the PLSR model gave a cross-validated R^2^ of 0.82 and RMSE of 51 mg m^−2^ for canopy Chl content in winter wheat, ranging between 38 and 475 mg m^−2^. Lu et al. [37] achieved significant prediction of the leaf Chl content of the three leafy green vegetable crops (pakchoi, Chinese white cabbage, and Romaine lettuce), using a combination of the PLSR method and red-edge region, as both combinations are insensitive to the difference between the adaxial and abaxial leaf structure. The PLSR combined with red-edge region exhibited the highest accuracy in estimating leaf Chl content of pakchoi, Chinese white cabbage, and Romaine lettuce with an R^2^ of 0.81, 0.89, and 0.83 and an RMSE of 62.44 mg m^−2^, 45.18 mg m^−2^, and 38.58 mg m^−2^, respectively. Because the type and number of input variables in multivariate regression methods significantly impacts their performance in the estimation of biochemical plant traits, it is still necessary to test the performance of different forms of SRIs in estimating leaf Chl content when they are coupled with such methods.

The primary goal of this study was to assess the relationship between canopy spectral reflectance measurements and the different units of measurements of Chl content (Chl _plant_, g plant^−1^; Chl _area_, μg cm^−2^; Chl _SPAD_, SPAD value) using different published algorithm forms of SRIs separately or coupled with PLSR and SMLR models. The SMLR method was used to extract the most important indices within each form of SRIs that explain the most variability in each unit of measurement of Chl content. The capability of different SMLR models based on these important indices in predicting the different units of measurement of Chl content was examined under various conditions (seasons, salinity levels, and genotypes).

## 2. Materials and Methods

### 2.1. Field Experimental Description

**Figure 1:**
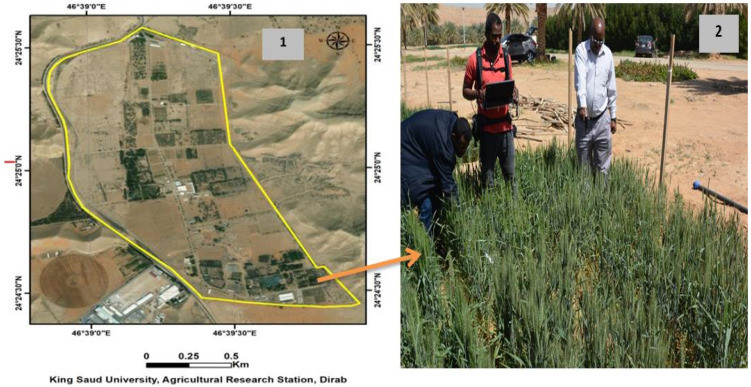
Location of the experimental field at the Research Station of the College of Food and Agriculture Sciences, King Saud University (**1**), and canopy spectral reflectance measurements (**2**).

The experiments of this study were conducted at the Research Station of King Saud University, Riyadh, Saudi Arabia (24°25′ N, 46°34′ E; elevation, 400 m) during 2017/2018 and 2018/2019 growing seasons (Figure 1). Two spring wheat genotypes (Sakha 61 and Sakha 93) were used in this study. Both genotypes were evaluated previously under control and field conditions and identified as salt-sensitive (Sakha 61) and salt-tolerant (Sakha 93) genotypes [50,51]. To evaluate both genotypes in simulated close-to-field conditions, a subsurface water retention technique (SWRT) was used as a growth platform. The advantages of using this technique as a growth platform when compared with the pot experiment and natural field conditions, and the setup of this technique, have been described in detail in our previous studies [6,52,53].

At the research station and during the growing season of spring wheat (from December to April), the rainfall ranged from 8.0 to 25.0 mm, the mean temperature ranged from 12.9 °C to 32.2 °C, and the humidity ranged from 17.7% to 47.5%. Soil texture is sandy loam with a pH of 7.85, bulk density of 1.48 g cm^−3^, organic matter of 0.46%, electrical conductivity of 1.12 dS m^−1^, available N of 45.2 mg kg^−1^, available P_2_O_5_ of 2.44 mg kg^−1^, and available K_2_O of 186.9 mg kg^−1^ [54].

### 2.2. Experimental Design, Agronomic Practices, and Salinity Treatments

The experiments were conducted in a split-plot design with three replications. The three salinity treatments, namely control (0.35 dS m^−1^), moderate salinity level (6.0 dS m^−1^), and high salinity level (12.0 dS m^−1^), and the two genotypes were assigned to the main plots, whereas the two genotypes were distributed randomly in subplots.

The seeds of each genotype were planted on 5 December 2017, and 2018, in sex 6 m-long rows spaced 0.2 m apart at a seeding rate of 150 kg ha^−1^. The two genotypes were fertilized with a nitrogen–potassium–phosphorus (NPK) fertilizer at a rate of 180 kg ha^−1^ of N, 60 kg ha^−1^ of P_2_O_5_, and 60 kg ha^−1^ of K_2_O. The entire amount of potassium (as potassium chloride, 50% K_2_O) and phosphorus (as calcium superphosphate, 18.5% P_2_O_5_) and 50 kg ha^−1^ of nitrogen (as ammonium nitrate) were applied before sowing. The plants were fertilized again at tillering and booting growth stages with 50 kg ha^−1^ of nitrogen.

The two genotypes were irrigated with non-saline water for the first two weeks after sowing in the three salinity treatments. Thereafter, the control treatment continued to be irrigated with non-saline water, while the treatments treated with moderate and high salinity levels were irrigated with artificial saline water containing 60 mM and 120 mM NaCl L^−1^ solution, respectively, until the final irrigation. The irrigation water was applied using a low-pressure surface irrigation system. This system consisted of a main line (76 mm in diameter) that delivers water from plastic water tanks (5.0 m^3^) to each subplot. To control the amount of water delivered to each subplot, this main line was branched off to the sub-main hoses at each subplot and equipped with a manual control valve. The genotypes were irrigated with fresh water 10 times in the control treatment, while in the two salinity treatments, they were irrigated with fresh water 2 times and with saline water 8 times. The irrigation frequency and rate was adjusted according to plant growth stage and environmental conditions.

### 2.3. Canopy Hyperspectral Reflectance Measurements

Canopy spectral reflectance was captured at the middle anthesis growth stage (Zadoks scale, ZS 65) [55], from two different places within each subplot within ±2 h of solar noon, under clear-sky conditions using ASD FieldSpec 4.0 spectroradiometer (Analytical Spectral Devices Inc., Boulder, CO, USA). This device is able to capture spectral reflectance from the canopy in the range between 350 and 2500 nm with a final band interval of 1 nm and spectral resolution ranging between 3 nm below 1000 nm and 10 nm between 1000 and 2500 nm. To efficiently capture the canopy spectral reflectance, the optical sensor of the device, which had a field of view of 25°, was held vertically approximately 0.8 m above the canopy in the nadir orientation. This situation for optical sensor enables it to cover an area of approximately 0.15 m^2^ of plant canopy. Before capture the spectral reflectance for the plants in each subplot, a white barium sulphate panel (Labsphere, Inc., North Sutton, NH, USA) was used to calibrate the device in order to avoid any changes in atmospheric conditions and sun irradiance on the spectral measurements [21]. The final canopy spectral reflectance curve was the average of four sequential measurements with 10 scans for each.

### 2.4. Spectral Reflectance Indices (SRIs)

Data of spectral reflectance were used to derive 83 published two- and three-band SRIs, using the wavelengths (λ1, λ2, and λ3) in the 400–850 nm regions (Table 1). These SRIs were selected as previous studies found that these SRIs were successfully used to estimate leaf Chl content in a number of plant species accurately. In this study, these SRIs were classified into four types of indices based on their algorithm forms: simple ratio index (SR), modified simple ratio (MSR), normalized difference (ND), and modified normalized difference (MND), as shown in the below equations:

SR = R_λ1_/R_λ1_

MSR = (R_λ1_/R_λ1_) − 1 or (1/R_λ1_) − (1/R_λ2_) or R_λ1_/(R_λ2_ + R_λ3_)

ND = (R_λ1_ − R_λ2_)/(R_λ1_ + R_λ2_)

MND = (R_λ1_ − R_λ2_)/(R_λ3_ + R_λ4_) or (R_λ1_ − R_λ2_)/(R_λ1_ − R_λ3_)

### 2.5. Chlorophyll Content Measurements

After spectral measurements, the leaf SPAD readings (Chl _SPAD_) were obtained from 10 fully expanded leaves, randomly selected from the spectral collection area using a chlorophyll meter (SPAD-502, Konika Minolta, Japan). The SPAD reading for each leaf was an average of three SPAD-502 readings, taken from three places from leaf base to apex. Additionally, to ensure consistency between the SPAD readings and leaf Chl content, quantitatively determined by a spectrophotometer in the laboratory, leaf discs were collected immediately after the SPAD-502 readings from an approximate position on the leaf sample at which the SPAD measurements were obtained. Ten leaf discs (~0.28 cm^2^ each) were taken by hole puncher and collected in a micro-centrifuge Eppendorf tube, immediately wrapped in aluminium foil and stored in ice. The samples were transported to the laboratory within 45 min and ground in the dark with 95% (*v*/*v*) ethanol using a clear mortar. Subsequently, the leaf pigment mixture was transferred to a 50 mL volumetric flask with 80% ethanol and one part of the homogenous solution was placed in a plastic tube and centrifuged for 20 min at 5000× *g*. The supernatant was transferred from a plastic tube to a cuvette to immediately measure its absorbance using a UV-VIS spectrophotometer (UV-2550, Shimadzu, Kyoto, Japan) at 645, 663, and 470 nm wavelengths. The total leaf Chl content was calculated using the formula provided by Lichtenthaler and Wellburn [56] and Wellburn [57]. Then, the total Chl content was converted into leaf Chl content per area (Chl _area_ μg cm^−2^) using the following equations:

Chl (mg g^−1^) = Chl (mg L^−1^) × Vol. solvent (mL)/1000/leaf fresh weight (g)

Chl (μg cm^−2^) = Chl (mg g^−1^) × 1000 × leaf fresh weight (g)/leaf area (cm^−1^)

The total Chl content per plant (Chl _plant_) was calculated from the total leaf area per plant (LA _plant_) and the Chl content per leaf area (Chl _LA_) using the following equation [58]:

Chl _plant_ = LA _plant_ × Chl _LA_

### 2.6. Statistical Analyses

To test the impacts of salinity levels, cultivars, and their interaction on different units of Chl content (Chl _area_, Chl _plan_, and Chl _SPAD_) during two growing seasons, the analysis of variance (ANOVA), appropriate for a randomized complete block split-plot design, was used. To compare the differences between the mean values of the three units of Chl content between salinity levels and genotypes, Duncan’s test at a *p* ≤ 0.01 and 0.05 significance level was applied. Pearson’s correlation coefficient (r) was calculated under each salinity level (*n* = 24), for each genotype (*n* = 36), and across all salinity levels and genotypes (*n* = 72) in order to evaluate the relationship between the three units of Chl contents. The relationship of 83 SRIs with each unit of Chl content across all conditions (salinity levels, genotypes, and seasons) was evaluated by simple linear regression.

Because the PLSR algorithm has an inferential capability, it was used in this study to model a possible relationship between different forms of SRIs (predictor variables) and each unit of Chl content (response variable). This algorithm is an effective method that specifies a relationship between a set of predictor variables and response variables. In this algorithm, the optimum number of latent variables (ONLVs) entering a final model needs to be carefully identified to represent the calibration data without excessive overfitting or under-fitting. The ONLVs were determined by employing a leave-one-out cross-validation (LOOCV) scheme. The best ONLV is that yielding the smallest root mean square error (RMSE) and the largest coefficient of determination (R^2^). Random 10-fold cross-validation was employed to the datasets from the two seasons to increase the robustness of the results. In this study, PLSR modelling was performed using Unscrambler 10.2 software (CAMO Software AS, Oslo, Norway).

To determine the most influential indices within each form of SRIs accounting for the highest variability in each unit of Chl content, the indices of each form of SRIs and three units of Chl content of pooled data (*n* = 72) were applied to SMLR as independent and dependent variables, respectively. To predict three units of Chl content under specific conditions (each of salinity level, genotype, and season), the different models of the best indices of each form of SRIs were used. The model with the lowest values of RMSE and the highest values of R^2^ of the linear relationship between the observed and predicted values of each unit of Chl content was designated the model with the higher prediction accuracy. The SMLR was performed using Sigma Plot for Windows (Version 22.0, SPSS, Chicago, IL, USA).

## 3. Results

### 3.1. Response of Different Units of Measurments of Chlorophyll Contents to Salinity Levels and Genotypes

As shown in Table 2, the control and moderate salinity levels (6 dS m^−1^) did not differ in Chl _SPAD_, while the other two units of Chl content (Chl _area_ and Chl _plant_) significantly decreased with increasing salinity levels. Averaged over the two seasons, decreases in both Chl units for 6 dS m^−1^ and 12 dS m^−1^ treatments relative to control treatment were 16.4% and 39.5% for Chl _area_, and 28.9% and 57.5% for Chl _plant_, while it was only 2.4% and 18.5% for Chl _SPAD_, respectively (Table 2). In addition, the two genotypes did not differ in Chl _SPAD_, while showing significant differences in the other two units of Chl contents, with the salt-sensitive genotype Sakha 61 always exhibited lower values for both units than those of the salt-tolerant genotype Sakha 93. Furthermore, the two genotypes did not differ in Chl _SPAD_ under the three salinity levels. However, averaged over the two seasons, the percentage reduction under 6 dS m^−1^ relative to control treatment reached 10.2 and 24.1% for Chl _area_, and 19.6 and 47.9% for Chl _plant_ for Sakha 93 and Sakha 61, respectively. The high salinity level was found to have resulted in decreases in Chl*t*_area_ of 31.6 and 48.6%, and Chl*t*_plant_ of 49.3% and 70.6% for Sakha 93 and Sakha 61, respectively, when compared with the control treatment (Table 2).

### 3.2. Relationships among Different Units of Measurements of Chlorophyll Contents under Salinity Levels and for Genotypes

The relationships among all units of Chl content were evaluated for each salinity level (*n* = 24), each genotype (*n* = 36), and when data for the salinity levels, cultivars, and seasons were pooled together (*n* = 72) (Table 3). All units of Chl content showed strong positive correlations with each other for pooled data (r = 0.77–0.94), as well as for Sakha 93 (r = 0.73–0.91) and Sakha 61 (r = 0.78–0.95). Contrarily, the Chl _SPAD_ showed no significant correlation with Chl _area_ and Chl _plant_ under control and moderate salinity level. However, for high salinity level, Chl _area_ was highly and significantly correlated with Chl _plant_ (r = 0.94), while the Chl _SPAD_ had a moderate correlation with Chl _area_ (r = 0.52) and Chl _plant_ (r = 0.62) (Table 3).

### 3.3. Relationship between Different Units of Measurements of Chlorophyll Content and Each Type of Spectral Reflectance Indices

**Figure 2:**
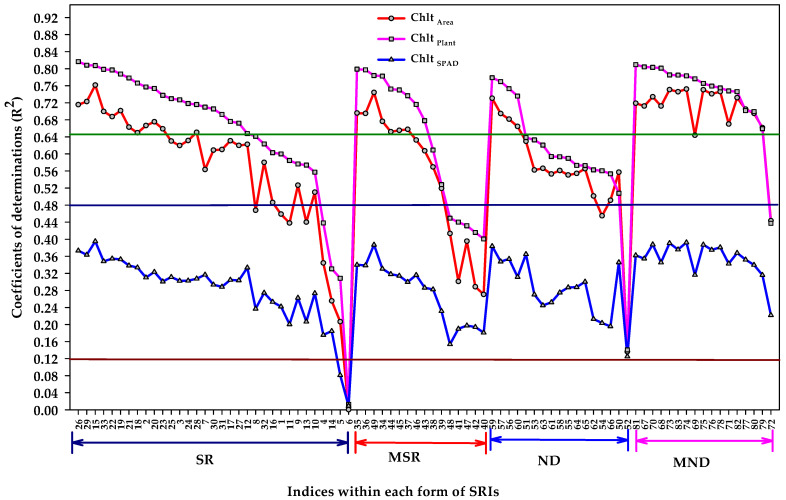
Coefficient of determinations (R^2^) for the linear relationships of indices within each form of spectral reflectance indices (simple ratio (SR), modified simple ratio (MSR), normalized difference (ND), and modified normalized difference (MND)) and the chlorophyll content based on area (Chl _area_), based on plant (Chl _plant_), and based on SPAD meter (Chlt _SPAD_). Estimates were calculated across all data (*n* = 72). R^2^ values ≥ 0.12 are significant at alpha = 0.05. The name and abbreviation of each number for different indices within each form of SRI is listed in Table 1.

Figure 2 shows the relationship of different units of Chl content with the indices of each form of SRIs. In general, the different forms of SRIs (SR, MSR, ND, and MND) showed the same pattern for their relationship with different units of Chl content, with nearly all indices of each form of SRIs estimated Chl _plant_ and Chl _area_ better than Chl _SPAD_. Nearly all indices of each form of SRIs exhibited a moderate to strong relationship with Chl _plant_ and Chl _area_ and a weak to moderate relationship with Chl _SPAD_ (Figure 2). Only very few indices exhibited nonsignificant relationship with the three units. One, one, and four out of the 33 SR form showed a nonsignificant relationship with Chl _area_, Chl*t*_plant_, and Chl _SPAD_, respectively. There are no indices from MSR, ND, and MND, showing a nonsignificant relationship with the three units of Chl content (Figure 2).

### 3.4. PLSR Models for the Estimation of the Different Units of Measurements of Chlorophyll Content

Table 4 summarizes the R^2^ and RMSE of calibration (Cal.) and 10-fold cross-validation (Val.) datasets of the PLSR models for estimating the different units of Chl content using the pooled data (*n* = 72). The PLSR models were determined based on the indices of each form of SRIs, as well as all indices of the four SRIs forms together. In general, the different PLSR models also provided a more accurate estimation for Chl _plant_ and Chl _area_ than for Chl _SPAD_ in both the Cal. and Val. datasets. In addition, all PLSR models generated a strong estimation performance in both Cal. and Val. datasets for Chl _plant_ (R^2^ ranged from 0.77 to 0.86), while they exhibited a strong estimation performance in Cal. datasets (R^2^ ranged from 0.65 to 0.82) and a moderate to strong estimation performance in Val. datasets (R^2^ ranged from 0.63 to 0.77) for Chl _area_. The different PLSR models showed a weak to moderate estimation performance in both Cal. and Val. datasets for Chl _SPAD_ (R^2^ ranged from 0.29 to 0.58) (Table 4). The PLSR models based on the indices of ND and MND forms, and the four forms together of SRIs in the Cal. datasets and MND form and the four forms together of SRIs in the Val. Datasets, were the best models to accurately estimate Chl _area_. The PLSR models based on the indices of all forms of SRIs separately or combined together were effective in accurately estimating Chl _plant_ in both Cal. and Val. datasets. The PLSR models based on the indices of ND form in both Cal. and Val. datasets were the best models to accurately estimate Chl _SPAD_ (Table 4).

### 3.5. Extraction of the Most Influential Indices within Each Form of SRI for Estimating the Different Units of Measurements of Chlorophyll Content

The indices of each form of SRIs were analysed by SMLR using the pooled data (*n* = 72) in order to extract the most influential indices within each form of SRIs that contributed the major variation for each unit of Chl contents. In general, the different SMLR models also provided a more accurate estimation for Chl _area_ (R^2^ ranged from 0.73 to 0.79) and Chl _plant_ (R^2^ ranged from 0.80 to 0.84) than for Chl _SPAD_ (R^2^ ranged from 0.39 to 0.43) (Table 5). The RGI-2, FRI-2, PARS-a, and BGI-3 were considered as the most influential indices within SR form and accounted for 78%, 84%, and 43% of the variation in Chl _area_, Chl _plant_, and Chl _SPAD_, respectively. A combination of RVSI and Chlb, RVSI and CI-2-D_Red-edge_, and RVSI were considered as the most influential indices within MSR form and accounted for 77%, 83%, and 39% of the variation in Chl _area_, Chl _plant_, and Chl _SPAD_, respectively (Table 5). Among the indices within the ND form, the combination of NDVI-5 and PSND-c or NDVI-5 and Lic-2 accounted for 80% and 43% of the variation in Chl _plant_ and Chl _SPAD_, respectively, while the NDVI-5 individually explained 73% of the variation in Chl _area_. Among the indices within the MND form, the combination of MDATT-2 and SIPI or MDATT-2 and MSR-2 explained 79% and 83% of the variation in Chl _area_ and Chl _plant_, respectively, as well as the MDATT-2, which individually explained 39% of the variation in Chl _SPAD_ (Table 5).

### 3.6. Validation of Predictive Models for Different Units of Measurements of Chlorophyll Content Based on Influential Indices Selected from Each Type of SRIs

The different models presented in Table 5 were applied to predict each unit of Chl content for each salinity level (Table 6), genotype, (Table 7) and season (Table 8). The results showed that the different predictive models for different types of SRIs failed to predict any unit of Chl content under the control treatment, as well as Chl _SPAD_ under moderate and high salinity levels, except the indices of ND and MND forms, which exhibited a weak relationship with Chl _SPAD_ under high salinity levels (Table 6). However, the different of SRIs exhibited a moderate to strong performance for predicting Chl _plant_ (R^2^ ranged from 0.56 to 0.82) and the Chl _area_ (R^2^ ranged from 0.54 to 0.74) under moderate and high salinity levels (Table 6).

In general, the different predictive models for different forms of SRIs provided a more accurate estimation of Chl _area_ and Chl _plant_ than they did for Chl _SPAD_ for both genotypes (Table 7). Additionally, the different forms of SRIs exhibited a strong and adequate performance for predicting Chl _area_ and Chl _plant_ for both genotypes, while they exhibited a weak to moderate performance for predicting the Chl _SPAD_ for both genotypes (Table 7).

For both seasons, in general, the different predictive models for different forms of SRIs provided a more accurate estimation of Chl _area_ (R^2^ ranged from 0.71 to 0.82) and Chl _plant_ (R^2^ ranged from 0.80 to 0.84) than they did for Chl _SPAD_ (R^2^ ranged from 0.33 to 0.55) (Table 8).

## 4. Discussion

Chlorophylls are one of the most important photosynthetic components that provide a key indicator of the photosynthetic potential of plants. Consequently, any reduction in leaf Chl contents would reduce the photosynthetic capacity of the plants, which eventually leads to a significant reduction in plant growth and productivity; more than 90% of crop biomass and yield are generated from photosynthesis [59]. Therefore, leaf Chl content, especially in stressed plants, could be considered as one of the fundamental criteria for the studies related to the evaluation of salt tolerance of genotypes to salt stress [7,58,59,60,61]. In this study, the response of three units of measurements of Chl content (Chl _area_, Chl _plant_, and Chl _SPAD_) to salt stress depended on salinity levels and the degree of salt tolerance of the two genotypes (Table 2). The Chl _area_ and Chl _plant_ substantially decreased as salinity levels increased, but the reduction in Chl _SPAD_ was observed only at high salinity levels. The two genotypes showed significant differences for Chl _area_ and Chl _plant_, but not for Chl _SPAD_, with the reduction in Chl _area_ and Chl _plant_ being lower in the salt-tolerant genotype than in the salt-sensitive ones under moderate and high salinity levels (Table 2). This result suggests that the ability of the salt-tolerant genotype to maintain a high content of Chl under salinity stress conditions could be considered, in general, as one of the biochemical indicators of salt tolerance in plants. Therefore, the total Chl content could be used as useful evaluating criteria for differentiating salt tolerance among wheat genotypes.

However, to understand the effect of salinity stress on total Chl content in plants, it is important to take into account the units that are used to express the content. Chl content per leaf area (μg cm^−2^), Chl content per leaf dry mass (g g^−1^), or Chl content per whole plant (g plant^−1^) are the most common units to quantify the amount of Chl in the leaf and whole plant [57,62]. Many previous studies have reported that salinity stress usually results in smaller and thicker leaves with a higher chloroplast density per unit leaf area, which can lead to an increase in Chl content when measured based on a single leaf and expressed in a unit of area [58,63,64]. However, when Chl content is measured based on the whole plant, the Chl content per plant decreases with increasing salinity levels as a result of smaller leaves [58]. This indicates that the interpretation of the relationship between different units of measurements of Chl content may not be stable if the salinity stress had significant impacts on leaf anatomy (size and thickness of leaf). Li et al. [65] reported that the increase in leaf thickness under salinity stress influenced the readings of the SPAD meter in a way that is independent of the impacts of salinity stress on Chl content. Therefore, to convert SPAD reading values into Chl content, the leaf thickness needs to be taken into account for the calibration of converted equations [66]. In this study, Chl _plant_ (g plant^−1^) was more correlated to Chl _area_ (μg cm^−2^) than to Chl _SPAD_ under each salinity level, for each genotype, or when the data of salinity levels and genotypes were pooled together (Table 3). Additionally, under high salinity levels (12 dS m^−1^), Chl _SPAD_ had non-significant correlation with Chl _area_ but it was significantly correlated with Chl _plant_ (Table 3). The reason why Chl _plant_ (g plant^−1^) was a more suitable unit to express plant Chl content than the other two units is probably because Chl _plant_ was obtained by using total leaf area per plant multiplied by Chl content per leaf area. Therefore, the Chl _plant_ takes into account the vertical heterogeneity of Chl content within wheat canopies.

### 4.1. Performance of Different Forms of Spectral Reflectance Index for Assessment of Different Units of Measurements of Chlorophyll Content

Previous studies have proposed several SRIs for the accurate estimation of Chl content using different algorithm forms of SRIs. To the best of our knowledge, very few studies have examined the performance of these SRIs for the estimation of Chl content based on different units of measurements. In this study, we examine the accuracy of Chl content estimates, which were expressed by different units of measurement, using different algorithm forms of SRIs (SR, MSR, ND, MND). As previously stated, because there are several physical and chemical canopy characteristics that affect the efficiency of canopy spectral reflectance for accurately estimating the plant traits of interest, the most important factors for determining the optimal SRIs are the formula and the sensitive wavelengths incorporated in this formula. The formula and wavelengths should be applied to a suitable algorithm to remove the effects of physical and chemical characteristics on canopy reflectance, as well as to increase the sensitivity of the SRIs to the plant traits of interest [20,28,37,67]. Most importantly, the effectiveness of different algorithm forms of SRIs for the accurate estimation of plant chemical compounds such as Chl and carotenoids contents depends also on the units that are used to express the measurements of these chemicals. For example, Yi et al. [68] found that the reciprocal index (1/R_515_ − 1/R_700_), which is in MSR form, performed better than the MND form (R_800_ − R_750_)/(R_750_ − R_670_) in estimating the carotenoids content expressed in g m^−2^ (r = 0.71), while the latter form showed the best correlation with carotenoids content expressed in μg cm^−2^ (r = 0.71) and mg g^−1^ (r = 0.67). Yi et al. [68] also reported that the most vegetation-related SRIs showed a stronger and stable correlation with carotenoids content expressed in μg cm^−2^ and g m^−2^ as compared with mg g^−1^. Kattenborn et al. [62] also concluded that quantification of plant pigments was performed better using remote sensing when it was expressed as leaf area-based content (μg cm^−2^) rather than as leaf mass-based concentration (g g^−1^). The results of this study found that nearly all indices of the four forms of SRIs estimated Chl content expressed as mg plant^−1^ (Chl _plant_) and μg cm^−2^ (Chl _area_) better than expressed as SPAD value (Chl _SPAD_). These indices exhibited a moderate to strong relationship with Chl _plant_ and Chl _area_ and a weak relationship with Chl _SPAD_ (Figure 2). These findings confirm that the units of measurement of Chl content play an important role in the efficiency of SRIs to accurately estimate Chl content. Furthermore, it is possible to upscale Chl content at the leaf level to the canopy level using reflectance measurements at the canopy level when the former level is expressed as μg cm^−2^ and the latter level is expressed as mg plant^−1^, by multiplying the Chl content at leaf level by total leaf area per plant. Similarly, the findings of Yi et al. [68] reported that the carotenoids content at leaf level and expressed as μg cm^−2^ or mg g^−1^ can be estimated at canopy level, using reflectance measurement at canopy level when it is expressed at canopy level as g m^−2^, by multiplying it by the total biomass of the plant.

### 4.2. Assessment of Different Units of Measurements of Chlorophyll Content Using a Combination of PLSR and Different Types of SRIs

As shown in Figure 2, some SRIs, especially within the SR and MSR forms, exhibited a weak correlation with the three units of measurements of Chl content. One reason for this might be that the algorithm of the different SRIs is always built based on a maximum of four sensitive wavebands, making it difficult to obtain a suitable index that increases the sensitivity of the index to Chl content, and at the same time, it has the ability to overcome the impacts of the different surface and internal structure of leaves on the performance of this index. For instance, photochemical reflectance index (PRI) exhibited a weak correlation with leaf Chl content because it is also sensitive to other leaf compounds, rather than to leaf Chl content [69]. In contrast, the Modified Datt (MDATT) index is recommended for the reliable estimation of leaf Chl content, as this index was generated from combinations of three wavelengths from the red-edge region to remove the effects of both adaxial and abaxial leaf surface structures [67]. Furthermore, due to the limited wavelengths in the algorithm of the different SRIs, several of them are usually saturated at high concentrations of Chl content, as well as at high LAI, which decreases their efficiency for the accurate estimation of Chl content [19,23,70]. Therefore, previous studies have recommended using a combination of several SRIs and PLSR models to improve the accuracy estimation of biochemical and biophysical traits, as this combination increases the number of wavelengths that are used in the prediction of the trait of interest [37,46,71,72,73]. Most of these studies, and other studies that are not mentioned here, found that PLSR coupled with several SRIs increased the accuracy of the estimation of different plant traits such as pigment contents, plant biomass, plant water content, and grain yield as compared with a single index. The results of this study showed that, in general, the PLSR models coupled with the four forms of SRIs, individually or combined, provided a more accurate estimation of Chl _plant_ and Chl _area_ than of Chl _SPAD_. Additionally, the PLSR models coupled with ND and MND forms or four forms together had the best performance in the estimation of the three units of measurements of Chl content in both the calibration and validation datasets (Table 4). The main reason for this finding might be that the MND form includes almost published MDATT indices (eleven indices). This type of index always demonstrated a better performance in the estimation of Chl content than other vegetation-related SRIs because it includes several wavebands that are insensitive to the structures of leaf surfaces and it also includes one band from each red, red-edge, and NIR region, which the three-band combinations were found to be suitable to use to accurately estimate leaf Chl content [28,37,67,74]. This may also explain why almost all indices of MDATT (ten out of eleven MDATT) in MND form exhibited a comparable performance for estimating the three units of Chl content using simple regression analysis, as did using the PLSR models coupled with MND form (compared data in Figure 2 and Table 4). This result agrees with Lu et al. [37], who concluded that the MDATT index was also effective for estimating the Chl content of leafy green vegetables accurately, however it was inferior to the PLSR models. This is because the PLSR models were based on all wavelengths in the red-edge region, which are not affected much by the differences between the abaxial and adaxial leaf structure.

### 4.3. The Performance of SMLR Models to Assess the Different Units of Measurements of Chlorophyll Content

Previous studies have reported that the same type of SRIs always exhibit high multicollinearity and over-fitting amongst themselves when using a simple statistical analysis, such as correlation and simple linear regression [75,76]. To avoid these problems, and to improve the performance of SRIs in the estimation of plant traits accuracy, SMLR models with a number of SRIs significantly improve the estimation of plant traits compared to a single SRI [77,78,79]. Huang et al. [80] found that the SMLR models provide useful information that can be used to obtain valuable information from leaf reflectance concerning leaf biochemistry. Interestingly, our results found that, in general, the different models of SMLR provided a more accurate estimation of Chl _plant_, followed by Chl _area_, than of Chl _SPAD_ (Table 5). Furthermore, although some SRIs exhibited a weak to moderate relationship with the three units of Chl content, such as RGI-2, BGI-3, Chlb, PSND-c, Lic-2, and SIPI (Figure 2), these indices are selected as the most influential SRIs in the SMLR models, explaining much of the variability in the three Chl content units (Table 5). These indices incorporated a combination of wavelengths from the spectrum regions (red, red-edge, and NIR regions) that are very sensitive to plant Chl content. In general, Chl absorption is strongly reduced in the red-edge and NIR regions compared to the red regions, and therefore the indices incorporated a combination of the wavelengths from the red region with wavelengths from the red-edge and/or NIR region increases their sensitivity to the canopy Chl content [21,23,31,35]. This may explain why such types of indices were effective for accurately estimating Chl content, especially when combined with SMLR models. Therefore, these influential indices extracted by the SMLR model were used to predict the three units of measurements of Chl content under each salinity level, and for each genotype and season (Table 6, Table 7 and Table 8).

In general, the ability of SMLR models for predicting the three units of Chl content depended on salinity level, genotypes, and seasons. The different predictive models of SMLR failed to predict the three units of Chl content under control conditions as well as Chl _SPAD_ under moderate and high salinity levels (except ND and MNDforms), whereas they exhibited moderate to strong relationships with Chl _plant_ (R^2^ = 0.56–0.82) and Chl _area_ (R^2^ = 0.54–0.71) under moderate and high salinity levels (Table 6). These results indicate that the ability of SRIs in the SMLR models for predicting the Chl content depended on the units of measurements of Chl content, the degree impacts of salinity stress on Chl content, and the degree of changes in Chl content under salinity stress. However, the forms of SRIs do not have any effect, as all forms of SRIs have almost the same performance for predicting chlorophyll content, particularly Chl _area_ and Chl _plant_, under moderate and high salinity levels.

The different models of SMLR provided a comparable performance for predicting the Chl _plant_ (R^2^ = 0.70–0.84) and Chl _area_ (R^2^ = 0.67–0.84) for both genotypes, while they provided a more accurate estimation of Chl _plant_ than they did for Chl _area_ for Sakha 61 and vice versa for Sakha 93 (Table 7). Additionally, these predictive models provided a more accurate estimation of Chl _SPAD_ for Sakha 61 (R^2^ = 0.36–0.48) than for Sakha 93 (R^2^ = 0.29–0.41) (Table 7). This result is believed to be associated with the substantial differences in Chl content between the two genotypes. The salt-tolerant sensitive genotype Sakha 61 may not have an efficient mechanism to protect their chlorophyll from degradation by salinity stress as did Sakha 93. This may generate a non-uniform distribution of Chl content within the canopy and the measured area in Sakha 61, which ultimately influences the spectral reflectance from the canopy, especially in the VIS and NIR regions [18,81]. Because the salinity stress may also generate significant variation in leaf internal structure, such as specific leaf mass and leaf thickness between genotypes [82], which ultimately influences SPAD meter readings, this may explain why the different predictive models provided a more accurate estimation of Chl _SPAD_ for Sakha 61 than for Sakha 93. Combined, this evidence indicates that the ability of SMLR models to predict different units of Chl content under salinity stress conditions is likely to be genotype-dependent.

## 5. Conclusions

This study evaluated the ability of different published algorithm forms of SRIs (SR, MSR, ND, MND) separately or combined with PLSR and SMLR models for estimating Chl contents that were expressed in different units at leaf level (Chl _area_ (μg cm^−2^) and Chl _SPAD_ (SPAD value)) and whole plant level (Chl _plant_ (mg plant^−1^)). The results allowed the following conclusions:

1- The different algorithm forms of SRIs showed the same pattern for their relationship with the three units of Chl contents, but the coefficients of determinations for Chl _plant_ and Chl _area_ were highly greater than those for Chl _SPAD_.
2- Nearly all MND forms were found slightly more efficient than other SRIs forms for estimating Chl _plant_ and Chlt _area_.
3- The PLSR models coupled with ND and MND forms, or four forms together, had the best performance in the estimation of the three units of measurements of Chl content, both in the calibration and validation datasets.
4- The indices that were extracted from each form of SRIs by SMLR explained 73–84% of the variability in Chl _area_ and Chl _plant_, and only 39–43% in Chl _SPAD_.
5- The ability of different models of SMLR for predicting the three Chl measurements depended on salinity levels, genotypes, and seasons.
6- Finally, our results indicate that the Chl content, measured on a laboratory basis at leaf level (Chl _area_), can be accurately estimated in a rapid and non-destructive manner using canopy spectral reflectance data, when the Chl content is also expressed in the whole plant (Chl _plant_).

## Figures and Tables

**Table 1:** Full names, abbreviation, and formulas of different types of newly constructed and published vegetation indices used in this study.

NO.	SRIs	Formula
	**Simple ratio (SR)**	
1	Simple ratio pigment index-1 (SRPI-1)	R_430_/R_680_
2	Simple ratio pigment index-2 (red-edge/green) (SRPI-2)	R_750_/R_556_
3	Simple ratio pigment index-3 (red edge/red) (SRPI-3)	R_750_/R_680_
4	Blue/Green pigment Index-1 (BGI-1)	R_400_/R_550_
5	Blue/Green pigment Index-2 (BGI-2)	R_420_/R_554_
6	Blue/Green pigment Index-3 (BGI-3)	R_450_/R_550_
7	Blue/Red pigment Index-1 (BRI-1)	R_400_/R_690_
8	Blue/Red pigment Index-2 (BRI-2)	R_450_/R_690_
9	Red/green pigment Index-1 (RGI-1)	R_690_/R_550_
10	Red/green pigment Index-2 (RGI-2)	R_695_/R_554_
11	Red/blue pigment Index (RBI)	R_695_/R_445_
12	RISPAD (SPADI)	R_650_/R_940_
13	Lichtenthaler index 1 (Lic1)	R_690_/R_440_
14	Fluorescence Ratio Index 1 (FRI-1)	R_690_/R_600_
15	Fluorescence Ratio Index 2 (FRI-2)	R_740_/R_800_
16	Carter index 1 (Ctr1)	R_695_/R_420_
17	Carter index 2 (Ctr2)	R_695_/R_760_
18	Carter index 3 (Ctr3)	R_750_/R_695_
19	Vogelmann red edge index 1 (VOG1)	R_740_/R_720_
20	Gitelson and merzlyak index 1 (GM-1)	R_750_/R_550_
21	Gitelson and merzlyak index-2 (GM-2)	R_750_/R_700_
22	Ratio vegetation index-1 (RVI-1)	R_750_/R_705_
23	Ratio vegetation index-2 (RVI-2)	R_800_/R_550_
24	Ratio vegetation index-3 (RVI-3)	R_800_/R_635_
25	Ratio vegetation index-4 (RVI-4)	R_800_/R_680_
26	Ratio analysis of reflectance spectra-a (PARS-a)	R_750_/R_710_
27	Ratio analysis of reflectance spectra-c (PARS-c)	R_760_/R_500_
28	Ratio analysis of reflectance spectra-c-D (PARS-c-D)	R_760_/R_515_
29	Ratio analysis of reflectance spectra-a-D (PARS-a-D)	R_780_/R_720_
30	Pigment Specific Simple ratio-a (PSSRa)	R_800_/R_675_
31	Pigment-specific simple ratio-b (PSSRb)	R_800_/R_650_
32	Pigment-specific simple ratio-c (PSSRc)	R_800_/R_470_
33	Datt derivative (DD)	R_850_/R_710_
**Modified Simple ratio (MSR)**		
34	red-edge chlorophyll index-1 (CI-1_red-edge_)	(R_750_/R_710_) − 1
35	red-edge chlorophyll index2-D (CI-2-D_red-edge_)	(R_760_/R_710_) − 1
36	red-edge chlorophyll index-3 (CI-3-_red-edge_)	(R_800_/R_710_) − 1
37	Green chlorophyll index (CI_green_)	(R_800_/R_550_) − 1
38	Carotenoid Reflectance Index-1 (CRI-1)	(1/R_510_) − (1/R_550_)
39	Carotenoid Reflectance Index-2 (CRI-2)	(1/R_510_) − (1/R_700_)
40	Anthocyanin (Gitelson) (AntGitelson)	R_780_(1/R_550_ − 1/R_700_)
41	Anthocyanin reflectance index 1 (Ant-1)	(1/R_550_ − 1/R_700_)
42	Anthocyanin reflectance index-2 (Ant-2)	R_800_(1/R_550_ − 1/R_700_)
43	Anthocyanin reflectance index-3 (Ant-3)	R_776_(1/R_530_ − 1/R_673_)
44	Ratio analysis of reflectance spectra-a (PARS-b)	R_675_/(R_650_*R_700_)
45	Ratio analysis of reflectance spectra-a (PARS-b)	R_675_/(R_640_*R_705_)
46	Chlorophyll a reflectance index a (Chla)	R_776_ (1/R_673_ − 1)
47	Chlorophyll b reflectance index b (Chlb)	R_776_(1/R_625_ − 1/R_673_)
48	Plant Senescence Reflectance Index (PSRI)	(R_680_ − R_500_)/R_750_
49	Red-Edge Vegetation Stress Index (RVSI)	0.5(R_722_ + R_763_) − R_733_
**Normalized difference (ND)**		
50	Normalized Phaeophytinization Index (NPQ)	(R_415_ − R_435_)/(R_415_ + R_435_)
51	Normalized Phaeophytinization-D Index (NPQ-D)	(R_482_ − R_350_)/(R_482_ + R_350_)
52	Photochemical reflectance index (PRI)	(R_531_ − R_570_)/(R_531_ + R_570_)
53	Photochemical reflectance index (PRI-D)	(R_531_ − R_580_)/(R_531_ + R_580_)
54	Normalized Pigment Chlorophyll Index (NPCI)	(R_680_ − R_430_)/(R_680_ + R_480_)
55	Normalized Difference Vegetation Index-1 (NDVI-1)	(R_750_ − R_680_)/(R_750_ + R_680_)
56	Normalized Difference Vegetation Index-2 (NDVI-2)	(R_750_ − R_705_)/(R_750_ + R_705_)
57	Normalized Difference Vegetation Index-3D (NDV3-D)	(R_780_ − R_715_)/(R_780_ + R_715_)
58	Normalized Difference Vegetation Index-4 (NDVI-4)	(R_800_ − R_670_)/(R_800_ + R_670_)
59	Normalized Difference Vegetation Index-5 (NDVI-5)	(R_800_ − R_550_)/(R_800_ + R_550_)
60	Normalized Difference Vegetation Index-6 (NDVI-6)	(R_800_ − R_700_)/(R_800_ + R_700_)
61	Normalized Difference Vegetation Index-7 (NDVI-7)	(R_850_ − R_680_)/(R_850_ + R_680_)
62	Pigment specific normalised difference-a (PSND-a)	(R_800_ − R_680_)/(R_800_ + R_680_)
63	Pigment specific normalised difference-b (PSND-b)	(R_800_ − R_635_)/(R_800_ + R_635_)
64	Pigment specific normalised difference-c (PSND-c)	(R_800_ − R_460_)/(R_800_ + R_460_)
65	Pigment specific normalised difference-c-D (PSND-c-D)	(R_800_ − R_482_)/(R_800_ + R_482_)
66	Lichtenthaler index 2 (Lic2)	(R_790_ − R_680_)/(R_790_ + R_680_)
**Modified normalized difference (MND)**		
67	Vogelmann red edge index-2 (VOG-2)	(R_734_ – R_747_)/(R_715_ + R_720_)
68	Vogelmann red edge index-3 (VOG-3)	(R_734_ – R_747_)/(R_715_ + R_726_)
69	Modified simple ratio of reflectance-1 (MSR-1)	(R_750_ – R_445_)/(R_705_ – R_445_)
70	Modified simple ratio of reflectance-2 (MSR-2)	(R_780_ − R_710_)/(R_780_ − R_680_)
71	Modified simple ratio of reflectance-3 (MSR-3)	(R_850_ − R_710_)/(R_850_ − R_680_)
72	Structure insensitive pigment index (SIPI)	(R_800_ − R_445_)/(R_800_ − R_680_)
73	Modified Datt index (MDATT-1)	(R_703_ − R_732_)/(R_703_ − R_722_)
74	Modified Datt index (MDATT-2)	(R_705_ − R_732_)/(R_705_ − R_722_)
75	Modified Datt index (MDATT-3)	(R_710_ − R_727_)/(R_710_ − R_734_)
76	Modified Datt index (MDATT-4)	(R_712_ − R_744_)/(R_712_ −R_720_)
77	Modified Datt index (MDATT-5)	(R_719_ − R_726_)/(R_719_ − R_743_)
78	Modified Datt index (MDATT-6)	(R_719_ − R_732_)/(R_719_ − R_726_)
79	Modified Datt index (MDATT-7)	(R_719_ − R_742_)/(R_719_ − R_732_)
80	Modified Datt index (MDATT-8)	(R_719_ − R_747_)/(R_719_ − R_721_)
81	Modified Datt index (MDATT-9)	(R_719_ − R_761_)/(R_719_ − R_493_)
82	Modified Datt index (MDATT-10)	(R_721_ − R_744_)/(R_721_ − R_714_)
83	Modified Datt index (MDATT-11)	(R_688_ − R_745_)/(R_688_ − R_736_)

**Table 2:** Effects of salinity levels, genotypes, and their interaction on different units of measurements of chlorophyll contents at the anthesis growth stage during two growing seasons.

Salinity Levels	Season 2017–2018			Season 2018–2019		
	Genotypes					
	Sakha 93	Sakha 61	Mean	Sakha 93	Sakha 1	Mean
Chlorophyll content based on area (Chl _area_, μg cm^−2^)						
Control	38.54 a	37.06 a	37.80 A	36.33 a	36.46 a	36.39 A
6 dS m^−1^	34.64 ab	28.43 bc	31.54 B	32.57 b	28.36 c	30.46 B
12 dS m^−1^	25.50 cd	19.53 d	22.52 B	25.72 c	18.96 d	22.34 C
Mean	32.90 A	28.47 B		31.53 A	27.93 B	
Chlorophyll content based on plant (Chl _plant_, mg plant^−1^)						
Control	11.88 a	10.78 a	11.33 A	12.92 a	11.26 a	12.09 A
6 dS m^−1^	9.63 a	6.54 b	8.09 B	10.39 a	6.74 b	8.56 B
12 dS m^−1^	5.85 b	3.70 c	4.78 C	6.55 b	3.79 c	5.17 C
Mean	9.12 A	7.01 B		9.95 A	7.26 B	
Chlorophyll content based on SPAD meter (Chl _SPAD_, SPAD value)						
Control	55.28 a	54.92 a	55.10 A	56.01 a	57.12 a	56.57 A
6 dS m^−1^	53.64 a	53.49 a	53.57 A	55.90 a	55.75 a	55.82 A
12 dS m^−1^	46.34 b	41.54 b	43.94 B	49.01 b	45.22 b	47.12 B
Mean	51.75 A	49.98 A		53.64 A	52.70 A	

Means followed by a different letter within a column are significantly different at *p* < 0.05 and 0.01 according to the Duncan’s test. Small letters indicate significant differences in the interaction between salinity level and genotype. Capital letters indicate significant differences among salinity levels or genotypes.

**Table 3:** Pearson’s correlation matrix between different units of measurements of chlorophyll contents for pooled data (*n* = 72), for each salinity level (*n* = 24), and for each genotype (*n* = 36).

Total Chlorophyll Parameters	1	2	3
Pooled data			
Chlorophyll content based on area (Chl _area_, μg cm^−2^) (1)	1.00	0.94 ***	0.81 ***
Chlorophyll content based on plant (Chl _plant_, mg plant^−1^) (2)		1.00	0.77 ***
Chlorophyll content based on SPAD meter (Chl _SPAD_, SPAD value) (3)			1.00
Control			
Chlorophyll content based on area (Chl _area_, μg cm^−2^) (1)	1.00	0.50 ^ns^	0.26 ^ns^
Chlorophyll content based on plant (Chl _plant_, mg plant^−1^) (2)		1.00	−0.04 ^ns^
Chlorophyll content based on SPAD meter (Chl _SPAD_, SPAD value) (3)			1.00
6 dS m^−1^			
Chlorophyll content based on area (Chl _area_, μg cm^−2^) (1)	1.00	0.73 ***	0.32 ^ns^
Chlorophyll content based on plant (Chl _plant_, mg plant^−1^) (2)		1.00	0.32 ^ns^
Chlorophyll content based on SPAD meter (Chl _SPAD_, SPAD value) (3)			1.00
12 dS m^−1^			
Chlorophyll content based on area (Chl _area_, μg cm^−2^) (1)	1.00	0.94 ***	0.45 ^ns^
Chlorophyll content based on plant (Chl _plant_, mg plant^−1^) (2)		1.00	0.62 **
Chlorophyll content based on SPAD meter (Chl _SPAD_, SPAD value) (3)			1.00
Salt-tolerant genotype Sakha 93			
Chlorophyll content based on area (Chl _area_, μg cm^−2^) (1)	1.00	0.91 ***	0.73 ***
Chlorophyll content based on plant (Chl _plant_, mg plant^−1^) (2)		1.00	0.76 ***
Chlorophyll content based on SPAD meter (Chl _SPAD_, SPAD value) (3)			1.00
Salt-sensitive genotype Sakha 61			
Chlorophyll content based on area (Chl _area_, μg cm^−2^) (1)	1.00	0.95 ***	0.84 ***
Chlorophyll content based on plant (Chl _plant_, mg plant^−1^) (2)		1.00	0.78 ***
Chlorophyll content based on SPAD meter (Chl _SPAD_, SPAD value) (3)			1.00

**, *** indicate significant at the 0.05, 0.01, and 0.001 probability levels, respectively. ^ns^ indicate not significant.

**Table 4:** Optimum number of latent variables (ONLVs), coefficient of determination (R^2^), root mean squared errors (RMSE) for calibration (R^2^_cal_ and RMSE_cal_), and ten-fold cross-validation (R^2^_val_ and RMSE_val_) statistics of partial least square regression models based on all indices within four forms of spectral reflectance indices (SRIs) for the assessment of chlorophyll content based on area (Chl _area_), based on plant (Chl _plant_), and based on SPAD meter (Chl _SPAD_). Estimates were calculated across all data (*n* = 72).

Chl Units	SRIs Forms	ONLVs	Calibration Dataset		Validation Dataset	
			R^2^_cal_	RMSE_Cal_	R^2^_val_	RMSE_Val_
Chl _area_	SR	4	0.73 ***	3.60	0.66 ***	4.08
	MSR	1	0.65 ***	4.09	0.63 ***	4.24
	ND	2	0.75 ***	3.46	0.73 ***	3.64
	MND	3	0.80 ***	3.10	0.77 ***	3.37
	All	11	0.82 ***	2.90	0.76 ***	3.43
Chl _plant_	SR	2	0.79 ***	1.44	0.77 ***	1.53
	MSR	2	0.79 ***	1.45	0.78 ***	1.52
	ND	3	0.83 ***	1.28	0.80 ***	1.43
	MND	3	0.83 ***	1.30	0.82 ***	1.39
	All	4	0.86 ***	1.19	0.82 ***	1.38
Chl _SPAD_	SR	1	0.31 ***	4.79	0.30 **	4.91
	MSR	1	0.32 ***	4.76	0.29 **	4.93
	ND	5	0.58 **	3.72	0.45 ***	4.29
	MND	1	0.35 ***	4.63	0.30 ***	4.84
	All	1	0.31 ***	4.77	0.30 ***	4.95

**, *** Significant at the 0.05, 0.01, and 0.001 probability levels, respectively. SR, MSR, ND, and MND indicate simple ration, modified simple ratio, normalized difference, and modified normalized difference SRIs types, respectively.

**Table 5:** Extraction of the most influential indices from each form of SRIs accounting for the major variation for chlorophyll content based on area (Chl _area_), based on plant (Chl _plant_), and based on SPAD meter (Chl _SPAD_) using stepwise multiple linear regression analysis. Estimates were calculated across all data (*n* = 72).

Measured Variables (y)	SRIs Groups	Best Fitted Equation	Model R^2^	Model RMSE
Chl _area_	SR	y = 134.66 − 5.33 (RGI-2) − 120.51 (FRI-2)	0.78 ***	3.29
	MSR	y = 17.02 − 1.53 (Chlb) + 418.88 (RVSI)	0.77 ***	3.36
	ND	y = −4.67 + 55.28 (NDVI-5)	0.73 ***	3.63
	MND	y = −65.88 − 8.81 (SIPI) + 63.10 (MDATT-2)	0.79 ***	3.23
Chl _plant_	SR	y = 29.43 − 31.86 (FRI-2) + 2.10 (PARS-a)	0.84 ***	1.31
	MSR	y = 2.36 + 1.94 (CI-2-D_Red-edge_) + 107.44 (RVSI)	0.83 ***	1.35
	ND	y = −2.98 + 36.56 (NDVI-5) − 14.78 (PSND-c)	0.80 ***	1.46
	MND	y = −8.14 + 18.07 (MSR-2) − 3.13 (MDATT-9)	0.83 ***	1.35
Chl _SPAD_	SR	y = 135.76 − 19.48 (BGI-3) − 90.06 (FRI-2)	0.43 ***	4.44
	MSR	y = 44.41 + 291.33 (RVSI)	0.39 ***	4.57
	ND	y = 27.75 + 55.21 (NDVI-5) − 14.80 (Lic-2)	0.43 **	4.44
	MND	y = −23.98 + 45.35 (MDATT-2)	0.39 ***	4.55

**, *** Significant at the 0.01, and 0.001 probability levels, respectively. SR, MSR, ND, and MND indicate simple ration, modified simple ratio, normalized difference, and modified normalized difference SRIs forms, respectively. R^2^ and RMSE indicate coefficient of determination and root mean squared error of the models, respectively.

**Table 6:** Function of linear validations between the observed and predicted values, coefficient of determination (R^2^), and root mean square error (RMSE) of linear regression models based on an individual selected spectral index (Table 5). These models were calibrated using a dataset of two seasons. Subsequently, the equations of calibration of distinct models (Table 5) were used to predict chlorophyll content based on area (Chl _area_), chlorophyll content based on plant (Chl _plant_), and chlorophyll content based on SPAD meter (Chl _SPAD_) for each salinity level (*n* = 24).

Measured Variables	SRIs Groups	Control			Moderate Salinity Level (6 dS m^−1^)			High Salinity Level (12 dS m^−1^)		
		Equation	R^2^	RMSE	Equation	R^2^	RMSE	Equation	R^2^	RMSE
Chl _area_	SR	y = 27.82 + 0.247x	0.09 ^ns^	1.67	y = 13.70 + 0.602x	0.71 ***	1.92	y = −1.43 + 0.961x	0.56 **	3.13
	MSR	y = 28.01 + 0.244x	0.13 ^ns^	1.64	y = 14.50 + 0.573x	0.74 ***	1.82	y = −3.97 + 1.054x	0.64 **	2.83
	ND	y = 41.47 − 0.126x	0.01 ^ns^	1.75	y = 15.15 + 0.559x	0.54 **	2.43	y = 1.68 + 0.818x	0.56 **	3.14
	MND	y = 33.52 + 0.093x	0.01 ^ns^	1.75	y = 15.67 + 0.525x	0.67 ***	2.06	y = −3.40 + 1.051x	0.64 **	2.84
Chl*t*_plant_	SR	y = 10.04 + 0.155x	0.02 ^ns^	1.18	y = 2.77 + 0.712x	0.71 ***	0.99	y = 7.79 + 0.849x	0.67 ***	0.89
	MSR	y = 9.94 + 0.163x	0.03 ^ns^	1.18	y = 2.97 + 0.691x	0.71 ***	1.00	y = −0.939 + 1.014x	0.75 ***	0.78
	ND	y = 14.10 − 0.199x	0.01 ^ns^	1.19	y = 3.60 + 0.595x	0.56 **	1.22	y = 0.621 + 0.740x	0.75 ***	0.78
	MND	y = 9.65 + 0.190x	0.02 ^ns^	1.18	y = 3.64 + 0.596x	0.69 ***	1.02	y = −0.904 + 1.005x	0.82 ***	0.66
Chl*t*_SPAD_	SR	y = 93.44 − 0.663x	0.07 ^ns^	2.16	y = 45.80 + 0.167x	0.03 ^ns^	2.91	y = 9.01 + 0.753x	0.15 ^ns^	4.32
	MSR	y = 71.08 − 0.264x	0.05 ^ns^	2.32	y = 45.47 − 0.174x	0.03 ^ns^	2.91	y = 9.01 + 0.749x	0.08 ^ns^	4.51
	ND	y = 133.74 − 1.380x	0.09 ^ns^	1.69	y = 52.93 + 0.029x	0.01 ^ns^	2.96	y = −12.95 + 1.205x	0.32 *	3.87
	MND	y = 119.89 − 1.063x	0.12 ^ns^	2.23	y = 50.10 − 0.079x	0.01 ^ns^	2.95	y = −26.11 + 1.349x	0.19 *	4.22

*, **, *** indicate significant at the 0.05, 0.01, and 0.001 probability levels, respectively. ^ns^ indicate not significant.

**Table 7:** Function of linear validations between the observed and predicted values, coefficient of determination (R^2^), and root mean square error (RMSE) of linear regression models based on an individual selected spectral index (Table 5). These models were calibrated using a dataset of 2 seasons. Subsequently, the equations of calibration of distinct models (Table 5) were used to predict the chlorophyll content based on area (Chl _area_), based on plant (Chl _plant_), and based on SPAD meter (Chl _SPAD_) for each genotype (*n* = 36).

Measured Variables	SRIs Groups	Salt-Tolerant Genotype Sakha 93			Salt-Sensitive Genotype Sakha 61		
		Equation	R^2^	RMSE	Equation	R^2^	RMSE
Chl _area_	SR	y = −0.54 + 1.004x	0.78 ***	2.41	y = −0.82 + 1.044x	0.76 ***	3.98
	MSR	y = −0.04 + 0.984x	0.81 ***	2.24	y = −1.80 + 1.086x	0.74 **	4.11
	ND	y = −5.54 + 1.156x	0.67 ***	2.96	y = −0.30 + 1.005x	0.73 ***	4.20
	MND	y = −2.26 + 1.057x	0.84 ***	2.06	y = −0.23 + 1.024x	0.75 ***	4.06
Chl*t*_plant_	SR	y = −0.17 + 1.016x	0.78 ***	1.27	y = 0.06 + 0.993x	0.84 ***	1.38
	MSR	y = −0.08 + 1.004x	0.78 ***	1.25	y = −0.02 + 1.006x	0.82 ***	1.47
	ND	y = −1.35 + 1.137x	0.70 ***	1.46	y = −0.02 + 1.006x	0.83 ***	1.44
	MND	y = −1.29 + 1.122x	0.80 ***	1.21	y = 0.29 + 0.978x	0.83 ***	1.46
Chl*t*_SPAD_	SR	y = 4.90 + 0.903x	0.41 **	3.36	y = −4.71 + 1.098x	0.42 **	5.31
	MSR	y = 10.74 + 0.795x	0.35 *	3.54	y = −9.91 + 1.198x	0.40 **	5.43
	ND	y = 11.89 + 0.776x	0.29 *	3.71	y = −5.63 + 1.111x	0.48 **	5.05
	MND	y = −7.38 + 1.054x	0.40 **	3.39	y = −9.34 + 1.101x	0.36 *	5.59

*, **, *** indicate significant at the 0.05, 0.01, and 0.001 probability levels, respectively.

**Table 8:** Function of linear validations between the observed and predicted values, coefficient of determination (R^2^), and root mean square error (RMSE) of linear regression models based on an individual selected spectral index (Table 5). These models were calibrated using a dataset of 2 seasons. Subsequently, the equations of calibration of distinct models (Table 5) were used to predict chlorophyll content based on area (Chl _area_), based on plant (Chl _plant_), and based on SPAD meter (Chl _SPAD_) for each season (*n* = 36).

Measured Variables	SRIs Groups	First Season			Second Season		
		Equation	R^2^	RMSE	Equation	R^2^	RMSE
Chl _area_	SR	y = −1.89 + 1.074x	0.82 ***	2.275	y = 1.56 + 0.938x	0.75 ***	2.527
	MSR	y = −1.18 + 1.055x	0.81 ***	2.433	y = 0.82 + 0.957x	0.75 ***	2.215
	ND	y = −2.30 + 1.079x	0.75 ***	2.148	y = 1.99 + 0.931x	0.71 ***	2.609
	MND	y = −2.09 + 1.080x	0.79 ***	2.410	y = 1.58 + 0.939x	0.80 ***	2.400
Chl*t*_plant_	SR	y = −0.17 + 1.004x	0.84 ***	1.505	y = 0.22 + 0.988x	0.83 ***	1.469
	MSR	y = −0.21 + 1.010x	0.83 ***	1.517	y = 0.27 + 0.983x	0.82 ***	1.468
	ND	y = −0.08 + 1.000x	0.80 ***	1.619	y = 0.12 + 0.995x	0.80 ***	1.386
	MND	y = −0.19 + 1.008x	0.82 ***	1.556	y = 0.23 + 0.989x	0.83 ***	1.544
Chl*t*_SPAD_	SR	y = −15.38 + 1.280x	0.55 **	2.638	y = 15.18 + 0.723x	0.33 *	2.389
	MSR	y = −9.42 + 1.177x	0.43 **	2.903	y = 11.39 + 0.789x	0.33 *	2.132
	ND	y = −8.09 + 1.151x	0.48 **	2.356	y = 10.69 + 0.802x	0.36 *	1.531
	MND	y = −15.85 + 1.204x	0.42 **	2.895	y = 4.62 + 0.854x	0.35 *	2.168

*, **, *** indicate significant at the 0.05, 0.01, and 0.001 probability levels, respectively.

## Data Availability

All data are presented within the article.
